# Efficacy of Acetylcholinesterase Inhibitors on Cognitive Function in Alzheimer’s Disease. Review of Reviews

**DOI:** 10.3390/biomedicines9111689

**Published:** 2021-11-15

**Authors:** Marta Pérez-Gómez Moreta, Natalia Burgos-Alonso, María Torrecilla, José Marco-Contelles, Cristina Bruzos-Cidón

**Affiliations:** 1 Preventive Medicine and Public Health Department, Faculty of Medicine and Nursing, University of the Basque Country UPV/EHU, 48940 Leioa, Spain; natalia.burgos@ehu.eus; 2 Pharmacology Department, Faculty of Medicine and Nursing, University of the Basque Country UPV/EHU, 48940 Leioa, Spain; maria.torrecilla@ehu.eus; 3 Laboratory of Medicinal Chemistry, Institute of Organic Chemistry (CSIC), Juan de la Cierva, 3, 28006 Madrid, Spain; jlmarco@iqog.csic.es; 4 Nursing I Department, Faculty of Medicine and Nursing, University of the Basque Country UPV/EHU, 48940 Leioa, Spain; cristina.bruzos@ehu.eus

**Keywords:** Acetylcholinesterase inhibitors, Alzheimer’s disease, cognition, Overview of reviews

## Abstract

Alzheimer’s disease (AD) is the most common form of dementia over the age of 65. It is estimated that 115.4 million people will be affected by AD by 2050. Acetylcholinesterase inhibitors (AChEI) are the only available and approved treatment for AD. The aim of the present study was to analyse the evidence on the efficacy of the AChEI in the treatment of cognitive symptoms of Alzheimer’s disease. For that purpose, a review of review of the systematic reviews (SRs) on this topic was carried out by Web of Science, PubMed, and The Cochrane Library, among others, were searched until 24 September 2021. Thirteen of the 1773 articles evaluated the efficacy of AChEI on cognitive function and/or general condition and/or behavioural disturbances of patients with mild to moderate AD. Methodological quality and risk of bias were rated using the ROBIS scale. The quality of the identified studies was high for nine of them, unclear for two, and finally only in two of the 13 studies did we detect low quality. Overall, AChEI showed very low efficacy in improving cognition in patients with mild to moderate AD. Better results were obtained in improving global state, with donepezil being the most effective treatment. No improvements in behavioural disturbances were found. Few high-quality reviews provide clear evidence of the effects of AChEI on cognition, global change, behaviour, and mortality. The data suggest that AChEI stabilize or slow cognitive deterioration, improving global status. In addition, data indicate that the use of AChEI decreases mortality in patients with mild to moderate AD. However, there is no evidence that they improve patient behaviour. Donepezil is the best therapeutic alternative at a dose of 10 mg/day.

## 1. Introduction

Alzheimer’s disease (AD) is the most common cause of dementia in people over 65 years of age and there is no effective treatment to date. AD is defined by the World Health Organization as a neurodegenerative disease of unknown etiology characterized by a progressive deterioration of memory and cognitive function [1]. It affects about 10% of people over 65 years of age [2], being more prevalent in women [3]. It is estimated that by 2050, 115.4 million people will be affected by this pathology [4] and it is among the leading causes of death worldwide [5]. AD is characterized by a prolonged duration, which makes it difficult to identify the factors associated with the onset of the pathology. In this regard, only 1–5% of cases have a known origin, with the origin of the disease being exclusively attributed to genetic or hereditary factors.

At the cellular level, AD is characterized by the appearance of so-called senile plaques, formed β-amyloid protein (βA) accumulation, suggested as the initial factor in the disease [6]. Another histopathological feature of AD is the formation of neurofibrillary tangles, formed by the accumulation of hyperphosphorylated Tau proteins at the intracellular level affecting axon stability and neuronal survival [7,8]. On the other hand, the progression of the disease itself causes an overwhelming loss of cholinergic synapses, which is the main cause of dementia [6]. It has been observed that the alterations of the cholinergic system start in the hippocampus and temporal cortex affecting learning, memory, thinking and sequencing in the mildest stage (cognitive function). It progresses to the temporal, frontal, and occipital lobes in the moderate stage affecting speech, comprehension, attention, language, spatial relationship in the moderate stage and finally, to the autonomic nervous system, affecting motor systems in the most advanced stage of AD [9].

The pharmacological treatments currently known for AD try to palliate the symptoms and reduce the progression of the disease by acting on the cholinergic system. The only drugs approved by the European Medicines Agency are the Acetylcholinesterase inhibitors (AChEI) donepezil, galantamine and rivastigmine, and memantine, a non-competitive N-methyl-D-aspartame glutamate receptor antagonist. The monoclonal antibody Aducanumab has recently been approved. It is a human monoclonal antibody selectively targets aggregates βA [10]. In terms of the efficacy, these treatments have been shown to be most effective in increasing cholinergic neurotransmission and reducing βA protein deposits, especially in mild to moderate AD. However, AChEI have been associated with several side effects such as nausea, vomiting, diarrhoea, abdominal pain, anorexia, headache, insomnia, muscle cramps, bradycardia, and syncope [11]. Since the efficacy of AChEI is arguable and tolerability may be low, the risk-benefit relationship of these interventions is unclear. 

There are currently more than 200 Systematic Reviews (SRs) and more than 1700 articles on AD and AChEI in PubMed, a fact that highlights the large amount of information available to the scientific community, and justifies the need to carry out a work that summarises the evidence of the efficacy of these drugs in the treatment of AD. This means a review of reviews, adding the level of quality to each one of them and thus generating a useful tool for the health professional. The present review aims to summarise the evidence on the efficacy of AChEI on cognitive function using the Alzheimer’s Disease Assessment Scale-Cognitive (ADAS-Cog) and Mini Mental State Examination (MMSE) scale. In addition, as secondary objectives, to summarise the evidence on the efficacy of AChEI on the general state of the patient using the Clinician Interview-Based Impression of Change, plus carer interview (CIBIC-Plus) scale and on behavioural alterations using the Neuropsychiatric Inventory (NPI) scale.

## 2. Materials and Methods

This review has been registered in the Prospective International Register of Systematic Reviews, PROSPERO: CRD42021250787.

The Preferred Reporting Items for Systematic Reviews and Meta-analysis (PRISMA) guideline [12] were followed (see Supplementary Materials: Table S1).

### 2.1. Review Questions

The responses to the PICO question in the review of reviews were:

P (Patients): Alzheimer’s patients. I (Intervention): AChEI (donepezil or rivastigmine or galantamine or tacrine), at all marketed doses, in any pharmaceutical form. C (Comparison): Double-blind randomised clinical trials (RCTs) versus placebo or versus another intervention. Treatment duration was a minimum of 12 weeks and no maximum limit. Comparison groups are placebo. O (Outcome, outcomes): Measures of progression of dementia based on assessment of cognitive status using standardised cognitive tests ADAS-Cog and MMSE. Their general assessment using the CIBIC-Plus, and their behaviour disturbances using the NPI.

### 2.2. Data Sources and Search Strategy

The search was carried out on 24 September 2021.

Searches in the main health databases: MEDLINE, EMBASE, CINAHL, PsycINFO and Lilacs (through Web of Science and Ovid SP). Search of The Cochrane Library, in PubMed, and Search of various gray literature sources, such as Epistemonikos and ResearchGate.

A search was carried out to identify SRs and meta-analyses (MA) published up to 24 September 2021 (no start date was given in order to cover as many publications as possible, taking into account that tacrine was withdrawn in September 2005 [13]) that evaluated the efficacy of AChEI in mild to moderate AD. The following keywords were used: “Alzheimer” or “Alzheimer’s disease”, “acetylcholinesterase inhibitors” or “donepezil” or “rivastigmine” or “galantamine” or “tacrine”, “MMSE”, “ADAS-Cog. “or “MMSE” or “ADAS-Cog.” and “systematic review”. The search strategy was detailed in the Supplementary Materials (Table S2. Search strategies).

We included SRs and MA of interventions with AChEI in patients with mild to moderate AD that at least measured cognitive function using the MMSE or the ADAS-Cog, and/or included the global assessment by an independent physician based on a clinical interview with the patient and caregiver (CIBIC-Plus) and/or assessed behavioural disturbances using the NPI.

### 2.3. Exclusion Criteria

We excluded all reviews that did not consider the patient cognitive status as measured by one of the two established scales, MMSE and/or ADAS-Cog, conference abstracts, or abstracts of publications without data and all reviews that independently analysed each AChEI. Publications not written in English, French, Italian, Portuguese, or Spanish were also excluded.

### 2.4. Screening Review (Review Selection) and Data Collection

Two overview authors independently (MP-GM, NB-A) screened for possible inclusion all identified SRs and MA that evaluated the effects of the above interventions. We assessed selection according to the review methodology, manually, to ensure that those with the appropriate population and measurement of the stated outcomes were selected for inclusion. Any disagreements were resolved by discussion with a third overview author (CB-C).

The methodology for data collection and synthesis was based on Chapter 22, “Overview of reviews” of the Cochrane Handbook of Systematic Reviews of Interventions [14].

Microsoft Excel 2019 software (Microsoft corporation, Redmond, Washington, EE.UU.), was used to create the different data disaggregation tables for the articles included in this work and Endnote software (Clarivate Analytics, Boston, Massachusetts, EE.UU.) was used as bibliographic manager.

### 2.5. Data Extraction and Data Measurement

Two of the overview authors independently (MP-GM, NB-A) extracted data from each SRs and MA and manually checked for compliance with the inclusion criteria (according to PICO question) for the intervention. Disagreements were resolved by consensus or discussion with a third review author (CB-C). Where information was missing from the review, published articles from the individual study were accessed and the authors of the SRs and/or MA were contacted for further details.

All the key information was extracted using two tables. On the one hand, one table with descriptive information on the review analyses and the methodological characteristics such as the type and period of the review, objectives and questions, design (database search), inclusion/exclusion criteria, type, number of studies, and the number of participants included. On the other hand, another table was used to collect the results of each study (outcome measure (relative risk (RR), odds ratio (OR), mean difference (MD), standardised mean difference (SMD), weighted mean difference (WMD)), results, conclusions, assessment of risk of bias, MA methods, MA results, and publication bias). 

### 2.6. Assessment of the Methodological Quality of the Study and Grading of the Evidence 

The methodological quality of the included SRs was assessed for relevance, by checking the population, intervention, comparator and review results and overview, independently using the ROBIS tool for assessing risk of bias in SRs [15]. The studies were checked by the two editors of the paper (MP-GM, CB-C). Differences were resolved by discussion and consensus and if necessary, a third reviewer was called in (NB-A).

The ROBIS tool, for the assessment of risk of bias in SRs, is aimed at four broad categories, among which interventions, and specifically review of reviews, stand out: 1. Assessing relevance (which is optional). 2. Identifying concerns in the review process and a third phase. 3. Judging the risk of bias. These three items point out aspects of the design of the review under review related to the potential for bias and are intended to help us judge the risk of bias in the review process, the results, and the conclusions. Using these indicators, the risk of bias is classified as high, low, or unclear.

To date this review of reviews is the first study to address the summary of the evidence of efficacy of cholinesterase inhibitors for the treatment of moderate to severe AD. This is high-quality and reliable material, as a starting point for the development of new therapeutic alternatives and as contrasted scientific material for all medical teams that need scientific evidence to carry out their daily work.

### 2.7. Quality Analysis of Reviews

The objective was to summarise and present the evidence provided by the SRs and MA over all these year analyses using quality criteria (ROBIS). No statistical analysis was performed.

## 3. Results

The search strategy described above provided 1836 eligible studies. Thus, the search in PubMed, the Cochrane Library and Web of Science resulted in 173, 57, and 1606 references, respectively. After removing the duplicates [16], 1773 studies were suitable for review and by reading the title and abstract 121 publications were considered candidates for analysis in accordance with the previously established inclusion criteria. After a review of the full text 13 articles that met all the inclusion criteria were selected for the analysis (Supplementary Materials: Table S3. Articles included in the review).

**Figure 1:**
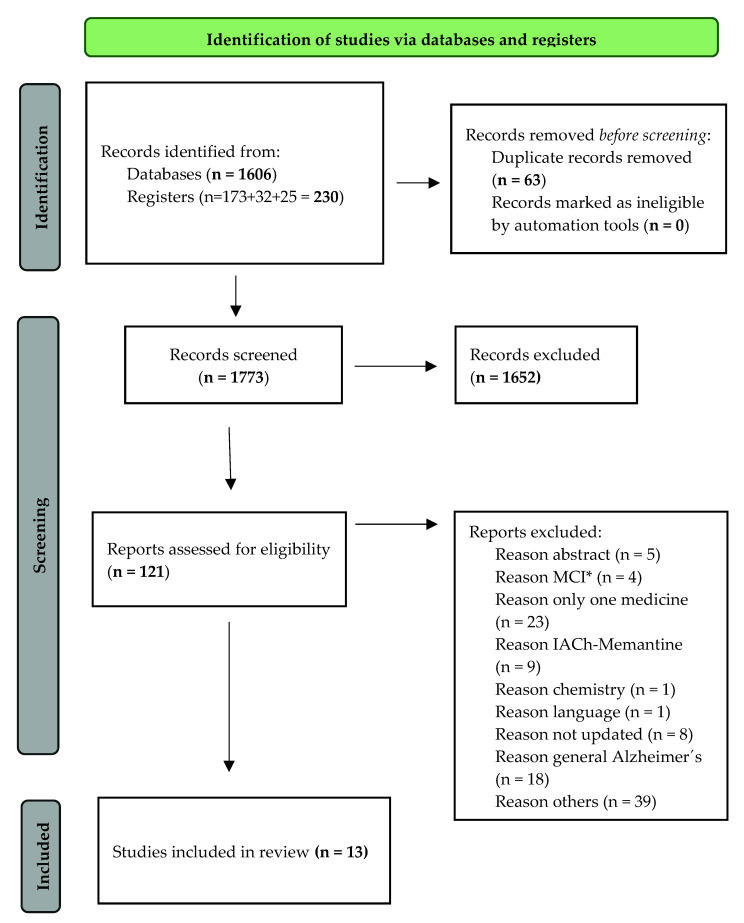
Preferred Reporting Items for Systematic Reviews and Meta-Analyses (PRISMA) [12] flow diagram. Search algorithm. * Mild Cognitive Impairment.

As shown in Figure 1, the PRISMA flowchart summarises the results obtained through the search strategy used in this review of reviews [12,17].

### 3.1. Review Characteristics of the Reviews

The 13 SRs included in this paper [10,17,18,19,20,21,22,23,24,25,26,27,28] include randomised clinical trials (RCTs). Eight of them [10,17,18,21,22,24,26,28] were systematic reviews with meta-analysis (SRMA), Supplementary Materials: Table S4. Characteristics of the analyses of the Systematic Reviews and Meta-Analyses. All these 13 studies [11,18,19,20,21,22,23,24,25,26,27,28,29] addressed the cognitive status of patients through the ADAS-Cog scale [11,18,19,20,22,23,24,25,26,27,28] and the ADAS-nonCog scale [21,29]. Seven studies [11,18,19,20,26,27,28] also assessed cognitive symptoms using the MMSE. Finally, ten studies assessed, in addition to cognition, the impression of the patient’s evolution by clinical interview [11,18,19,20,22,23,24,25,26,27], with the CIBIC-Plus scale and ten studies assessed behaviour changes with the NPI [11,18,19,21,22,23,24,25,28,29].

Ten of the 13 SRs [11,18,19,20,22,23,24,25,27,28] studied the efficacy of donepezil, rivastigmine, and galantamine compared to placebo with the exception of Livingston et al., 2000 [26], which did not study galantamine because it was not yet marketed and Grimmer et al., 2006 [21] and Trinh et al., 2003 [29] which did not evaluate rivastigmine. Three of the 13 studies [21,26,29] included tacrine in their studies. Three SMRs [19,22,25] and one SR [28] included comparisons between two AChEI in their studies. Only one SRMA [19], included comparisons of AChEI with memantine.

All the 13 studies used the MEDLINE and EMBASE databases, and with the exception of Livingston et al., 2000 [26], also the Cochrane Library. Eight of the 13 studies [11,18,19,20,22,23,27,28] very extensively searched the different databases referenced in Supplementary Materials: Table S4.

The inclusion criteria used by all studies were placebo-controlled RCTs, in four studies [19,22,25,28] also against another AChEI and in one [19] also against memantine. Only seven of the 13 [11,22,24,25,26,27,29] mentioned that they were double-blind, in mild to moderate AD diagnosed according to ICD-10 [30], DSM-5 [31], NINCDS-ADRDA [32] criteria, for at least 12 weeks. 

The number of trials included in the reviews ranged from 43 RCTs [33] to 5 RCTs [26] both with very good evidence and low risk of bias. The number of patients evaluated ranged from 16,106 in [18] to 1312 in [26]. 

In Supplementary Materials: Table S5. Summary of evidence. Effectiveness of the interventions analysed.

In six of the 13 studies [11,18,19,20,22,24,28] risk of bias was assessed. Two studies [11,18] used the Cochrane Collaboration Scale [14], one [20] the Jadad scale [34] and three [19,22,28] assessed attrition bias [14] and used modified NHS quality criteria [35].

The outcome measures for the eight MA were standardised mean difference (SMD) in six [11,18,19,24,25,29], weighted mean difference (WMD) in four [19,22,23,29], Odds Ratio (OR) with 95% Confidence Interval (95% CI) in four studies [11,18,25,27], Relative Risk (RR) with 95% CI in two studies [22,26] the latter also defined number needed to treat (NNT), absolute relative risk (ARR) and relative risk reduction (RRR). Kobayashi et al., 2016 [25] defined outcomes according to the heterogeneity values (I_2_) resulting from the MA. Three SRs [20,21,28] did not give outcome measures and were defined in Supplementary Materials: Table S5 as NA (not applicable).

Out of eight MA [10,17,18,21,22,24,26,28], only five [18,19,22,25,29] detected publication bias using funnel plots [36] and another one [29] also applied Kendall’s Tau correlation coefficient.

Seven articles [18,19,20,23,24,25,28] concluded that the AChEI donepezil, galantamine, rivastigmine and tacrine have little or poor risk-benefit ratio in improving cognitive status and overall patient outcome by clinical interview (Supplementary Materials: Table S5). Of these seven articles, five [18,19,23,25,28] showed very good quality and low risk of bias (Supplementary Materials: Table S6). Four more articles [11,22,26,27] assessed efficacy on cognition and patient global state, and only one study [11] showed poor quality and therefore high risk of bias according to the ROBIS tool. These four studies [11,22,26,27] claimed that AChEI are effective in slowing cognitive decline, global state and behaviour in mild/moderate AD.

With regard to efficacy on behaviour, two articles [21,29] confirmed an improvement in the behaviour and neuropsychiatric status of the AD patients; the study by Trinh N. et al., 2003 [29] classified as high quality and low risk of bias and the study published by Grimmer, T. et al. 2006 [21] classified as low quality and high risk of bias. The remaining eight articles [11,18,19,22,23,24,25,28] stated that the efficacy on behaviour was questionable and that further studies were needed. 

Finally, four studies [18,19,23,25] investigated the relationship between pharmacological treatment and mortality in AD patients. In the case of Kobayashi et al., 2016, they concluded a non-drug related mortality. However, Hyde et al., 2013 [23] Bond et al., 2012 [19] and Blanco-Silvente et al., 2017 [18] performed a MA, and only Blanco-Silvente et al., 2017 [11] showed a result in favour of decreased mortality (OR = 0.65) in AD patients treated with AChEI.

### 3.2. Study Quality and Evidence Synthesis

To address the quality of SRs the ROBIS scale was performed (Supplementary Materials: Table S6). Assessing the quality of reviews using ROBIS, a tool to assess risk of bias in SRs, presents the results of the assessment of the methodological quality of the included studies. Seven SRs included a MA [11,18,19,22,23,25,27,29] indicating a higher quality score, with a low risk of bias (smiley and green emoticon), providing high-quality causal evidence in their conclusions. Only one study [11] had a poor-quality score with a high risk of bias (sad and red emoticon). This was an RSMA conducted by a single reviewer and without any risk of bias assessment, neither of the included studies, nor in the results of the MA. Of the remaining studies, five were SRs [20,21,24,26,28] of which two contributed high-quality causal evidence and low risk of bias (smiley and green emoticon) [26,28], of the other three SRs [18,19,22] two contributed causal evidence of high quality and low risk of bias [22,26,28] two contributed causal evidence of unclear quality and unclear risk of bias (indifferent emoticon and grey) [21,24] and a final SR [20] scored poor quality, with a high risk of bias (sad emoticon and red). Two SRs [21,24], with 14 and 22 RCTs and 4625 and 8970 patients, respectively, scored unclear risk of bias, and the review was of unclear quality.

In summary (Table 1), nine articles [18,19,22,23,25,26,27,28,29] had a high grade of evidence and therefore a low risk of bias; only two [22,24] had an unclear risk of bias, as their grade of evidence was also unclear, and only two articles [11,20] from the literature had a low grade of evidence as they had a high risk of bias. 

## 4. Discussion

In the present study, all SRs and SRMA on the efficacy of AChEI in mild to moderate AD over the last two decades were reviewed. Most of the studies assessed the efficacy of the drugs used in the treatment of AD by measuring the cognitive evolution of the patients for at least 12 weeks. The assessment of cognition, global state based on the clinical interview and behaviour symptoms are three important variables to be considered, both in the evolution of the disease and in the efficacy of treatments for patients with AD [32,37]. For this purpose, the cognitive assessment scale, ADAS-Cog, was used in different studies. In addition, in seven of the reviewed studies [11,18,19,20,26,27,28] the MMSE was also evaluated. For of the ADAS-Cog scale, eight of the studies [11,19,22,23,25,26,27,29] suggested that the AChEI have a protective role on the cognitive function of AD patients. This was also supported by the seven studies that measured cognitive function with the MMSE scale. The assessment of global change based on the medical interview with the patient and caregiver, using the CIBIC-Plus test or scale, also supports the efficacy of AChEI in the treatment of AD patients. However, this efficacy on behaviour disturbances is not substantiated in most of the reviewed studies. 

Studies of high quality and low risk of bias, such as Blanco-Silvente et al., 2017 [18] (an MA that used Bayesian-framed meta-regression and included the largest sample size known so far), Bond et al., 2012 [19] (National Health Service, NHS England report) or Kobayashi et al., 2016 [25] included in this paper, statistically confirm the hypothesis of the efficacy of AChEI in mild to moderate AD but raise the importance of extrapolation to actual clinical efficacy. Measurement of cognition, global change, or behaviour reflect a benefit due to their therapeutic use and easy measurement, but there is an overestimation of the clinical effect. They are considered surrogate variables [16,38,39] that by themselves have no clinical value, but a benefit is attributed to them that should be compared to the (unknown) long-term risk of using the treatments. Therefore, these studies have also attempted to assess long-term clinical efficacy, quantifying mortality, symptom improvement, treatment discontinuation due to adverse events, or magnitude of effect. It is this additional evidence of clinical efficacy that shows a poor risk-benefit ratio of treatments and counteracts the better a priori results obtained in MA. There are reports that point out the poor benefit-risk ratio of these treatments, due to an overestimation of the real clinical benefit and a health risk that does not promote their administration, which justify the political decisions taken in favour of eliminating the funding of these treatments, as was the case in England and Wales in the period 2007–2010 [40] or in France since 2018 [41]. 

Using the results of the ADAS-Cog test to assess cognitive function makes it difficult to define and translate the improvement in cognitive symptoms into a therapeutic benefit. There is no clinical variable to measure function in AD patients [42]. The results obtained from the 17 MA [11,18,19,22,23,25,27,29] of ADAS-Cog and MMSE measurements for the different AChEI are modest. Only one study [23] of good-quality RSMA showed moderate statistical heterogeneity (I_2_ = 44.19%). If the intrinsic characteristics of the variable used are compounded by doubts about the robustness of the results described so far, the evidence for the efficacy of AChEI could be questioned. 

Regarding the improvement of global symptomatology, based on the impression of global change in the patient and caregiver medical interview, with CIBIC-Plus, for AChEI, significant differences in favour of the treatments were reported in seven of the ten studies [11,18,19,20,22,23,24,25,26,27] where global change was assessed, six [18,19,22,23,24,27] were high-quality MA with low risk of bias for the ROBIS tool [15]. The statistical significance obtained (*p* < 0.01), associated with the type of AChEI showed greater efficacy for donepezil versus galantamine and rivastigmine in longer treatments [18,19,23,25], with the derived hierarchy being donepezil > galantamine > rivastigmine [25]. The low statistical heterogeneity of the studies (I_2_ = 0) of high quality and low-risk-of-bias support the result [18,22], along with the studies by Raskind et al., 2004 [43] and Hashimoto et al., 2005 [44] suggesting that donepezil and galantamine have neuroprotective effects leading to disease delay. 

However, the improvement of neuropsychiatric symptoms measured with the NPI and assessed in high-quality MA studies with low risk of bias is unclear, as the statistical differences found cannot be estimated [19,23] or if estimated, are minimal [18]. Two pioneering studies [21,29], assessed behavioural disturbances only using the ADAS-nonCog and the NPI. Both studies describe a moderate or beneficial effect for AChEI in the ADAS-nonCog and NPI, respectively, as did Wang et al., 2015 [45]. Grimmer et al., 2006 [21,45] of low quality and high risk of bias and Trinh et al., 2003 [29], of high quality and low risk of bias, are two studies from the beginning of the century, and drug commercialization, two facts which deprives them of having temporal arguments to justify their claims. Later studies, however, contradict these results [18,19,23,25]. Kobayashi et al., 2016 [25], a high-quality, low-risk-of-bias MA on the ROBIS scale, clearly specified that AChEI should have significant efficacy for cognition and global change assessment, but efficacy on neuropsychiatric symptoms is questionable in patients with mild to moderate AD.

Kobayashi et al., 2016 [25] is the only article that was conducted by employees of Janssen pharmaceutical company; and was not affected by the sponsor bias [46,47] that many of the articles that have evaluated the efficacy of AChEI have. Kobayashi et al., 2016 [25] do not show positive results of the efficacy of AChEI on cognition, and even questioned the improvement in behavioural changes, thus supporting the results observed in the MA. However, other authors, such as Lockhart et al., 2011 [48], Matsunaga et al., 2014 [49] or Glinz et al., 2019 [50] reported a conflict of interest as they were subsidised by the companies, Pfizer, Janssen, or Novartis, among others, who marketed these treatments. All three studies [48,49,50] showed a benefit in support of AChEI in monotherapy, or in association with memantine. However, other authors [25,51], indicate the need for rigorously conducted comparative clinical trials to conclude a benefit in support of improved behaviour in AD patients.

At this point, two types of significance should be taken into account, statistical and clinical [52,53]. Moreover, to draw conclusions, the effect size must be combined with the volume of data. For this purpose, sequential trial analyses are used [54], a tool that considers both the number of patients included and the minimum expected effect of the intervention from a clinical point of view, providing further evidence. In the case in question, none of the SRs or SRMA suggested that the RCTs evaluated consider this type of significance. The existence of strict inclusion criteria in the inclusion of patients in the RCTs [55] together with a difficult extrapolation of results to clinical practice [33], prevent firm conclusions from being drawn.

As a relevant clinical variable, regarding the evolution of mild to moderate AD, three [18,19,23] of the studies encompassed in our work, together with Reisberg et al., 1996 [56], de Nordström et al., 2013 [57] and Kaushik et al., 2017 [58], suggested a reduction in mortality associated with the use of AChEI in mild to moderate AD, shedding some more light on the use of AChEI for AD.

Regarding the efficacy of AChEI on cognition, global change, and behavioural disturbances in AD patients, none of them showed positive results [59], i.e., no difference of 4 points in ADAS-Cog, no difference of 1 point in CIBIC-Plus, and no difference of 4 points in NPI was achieved.

It should be noted that the results derived from the present work have been assessed by quantifying the quality of the evidence, using the ROBIS risk of bias tool, which is recommended for this type of work [15]. A total of 70% of the included articles have a high quality and low risk of bias, 15% correspond to studies of unclear quality and unclear bias, and another 15% are included studies with low quality and high risk of bias. Within the 70% of studies with high quality and low risk of bias, one of the main RSMA articles summarising the evidence on the efficacy of AChEI for mild to moderate AD is included, Blanco-Silvente et al., 2017 [18].

### 4.1. Limitations and Strengths

This study has some limitations. First, the publication bias of SRMA and SRs could bias our result if not well described and not properly selected, but it does not seem to have influenced our case. We also must take into account the limitations affecting the analysis of the review of reviews as well as the limitations when performing the ROBIS and the possible attrition bias. On the other hand, the measurement of the MMSE, ADAS-Cog, CIBIC-Plus, or NPI scales may be a limitation if the same assessment scale is not used. Finally, another possible limitation is the loss of evidence from individual studies due to the overlap between reviews and the heterogeneity of the studies, which makes meta-analysis of the review of the reviews difficult.

To the best of our knowledge, the present work is the first large-scale investigation that summarises the evidence on the efficacy of AChEI drugs since their authorisation for the treatment of AD to date, presenting a novel and rigorous methodology.

### 4.2. Implications for Practice

The results of this work suggest that AChEI indicated for the treatment of AD, as almost the only therapeutic treatment, except for memantine and recently the Aducanumab, do not clinically improve the clinical symptoms of the disease.

We think that one of the main causes why known AChEI lack clinical efficacy is due to the fact that they have designed under the “one molecule–one target” therapeutic strategy, obviating thus the complexity of AD that would possibly require a new therapeutic approach, based on the concept and application of the “multitarget small molecules” (MSM) approach. This would mean that more efficient, single drugs for AD therapy should be able to bind, modulate, or inhibit simultaneously different biological targets involved in the progress and development of the disease. This means necessarily to select and refine the kind and number of the possible biological targets to tackle, by designing new ligands, for instance, by juxtaposition of suitable pharmacophoric groups responsible for the diverse appropriate biological/pharmacological properties that we wish to incorporate in our test molecules. The proof that this is not an easy task, is the fact that till now, despite the great effort dedicated to this goal, no such MSM has been reported. This is thus the challenge that we must deal with in the future for a more successful approach for the treatment of AD.

In this context, both the French Ministry of Health [41] and the National Health System of England and Wales [40], took the decision to withdraw them from funding due to a poor benefit-risk ratio, justifying this decision on the grounds of poor efficacy. One of the articles [19] included in this review changed the English government’s mind in favour of reinclusion in funding, due to a more favourable cost-effectiveness, although discrepancies continue to exist on this point [60]. Interestingly, it should be noted that although the drugs marketed so far do not improve the symptoms of AD, the study of new drugs with anticholinesterase and butyrylcholinesterase action remains key, since a deficit of ACh has been demonstrated in AD. On the other hand, future research should open the horizon towards the development of new pharmacological strategies to treat and cure the disease. The biochemists and molecular biologists, hoisting the flags of the Aβ and Tau hypotheses for AD, reached a stage, in an impressive effort, that has only recently resulted in the human monoclonal antibody Aducanumab [61], the first disease-modifying drug approved for AD. However, it is worth mentioning that during the review process, the FDA (US, 07 June 2021) approved the use of Aducanumab (Aduhelm, ^®^Biogen, Cambridge, MA, USA) for early phases of AD (56,000.00 USD/patient/year), despite an FDA advisory committee concluding that there is not enough evidence to support the effectiveness of the treatment. In our opinion, and after the extensive research work carried out to develop this work, future lines of research should focus on three main axes. First, new AChEI that delay the evolution of the disease, more efficient treatments are in progress based on super-AChEI designed under the “multitarget small molecule” umbrella [62]; secondly, studying epigenetics and designing drugs that are capable of modifying the human genome that cause AD [63,64] and, thirdly, the study of the human microbiota and how it influences the development of pathologies that trigger AD [65,66].

## 5. Conclusions

Positive results provided by several high-quality reviews over the last two decades suggest a stabilization or slowing of cognitive decline in mild to moderate AD by AChEI, but not an improvement in cognition as suggested or claimed in reviewed papers. Symptom improvements are slight, and treatments are also discontinued due to the appearance of adverse effects. Therefore, there is a poor benefit-risk ratio. Against this position, the results of this review of reviews are conclusive regarding the improvement of global symptomatology. AChEI improve the global condition of patients with mild to moderate AD. Donepezil represents the best therapeutic alternative for patients with mild to moderate AD by improving patient global condition (greater effects at 10 mg) and fewer adverse events occur than with rivastigmine or galantamine. There is a suggestion of a decrease in mortality in patients treated with AChEI versus placebo. There is no scientific evidence that AChEI improve the behaviour of patients with mild to moderate AD.

For the current clinical practice and health care professionals, the conclusion of this review of reviews is that unfortunately one of the approved therapies to treat AD patients, based on the administration of AChEI, has a limited effect on this pathology, but that no space for despair is allowed, as definitively renewed hopes for more efficient treatments are in progress based on super-AChEI designed under the MSM umbrella [62,67].

## Figures and Tables

**Table 1:** Summary of the risk of bias of the included studies according to ROBIS [Justification for concern: Low: 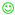, if all answers are yes or probably yes. High: 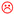, if any is no or probably no. Not clear: 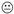, insufficient data].

Review ID	ROBIS Domain				Risk of Bias in the Review
	Domain 1: Study Eligibility Criteria	Domain 2: Identification and Selection of Studies	Domain 3: Data Collection and Study Evaluation Domain	4: Synthesis and Results	
Birks, JS. 2016 [11]	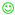	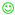	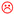	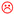	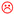 HIGH
Blanco-Silvente, L. et al. 2017 [18]	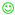	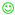	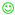	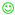	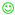 LOW
Bond, M. et al. 2012 [19]	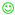	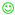	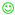	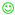	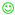 LOW
Clegg, A. et al. 2002 [20]	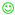	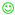	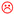	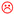	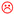 HIGH
Grimmer, T. et al. 2006 [21]	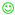	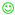	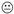	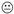	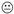 NOT CLEAR
Hansen, R. A. et al. 2008 [22]	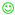	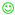	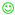	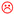	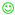 LOW
Hyde, C. et al. 2013 [23]	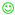	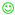	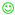	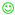	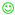 LOW
Kaduszkiewicz. H. et al. 2005 [24]	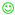	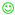	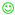	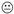	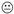 NOT CLEAR
Kobayashi, H. et al. 2016 [25]	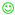	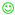	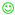	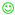	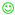 LOW
Livingston, G. et al. 2000 [26]	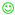	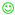	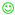	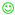	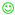 LOW
Ritchie, C. W. et al. 2004 [27]	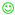	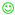	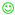	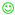	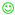 LOW
Takeda, A. et al. 2006 [28]	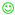	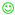	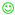	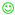	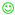 LOW
Trinh N. et al. 2003 [29]	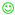	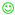	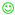	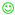	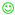 LOW

## Data Availability

Data is contained within Supplementary Material. The data presented in this study are available in Supplementary Materials.

## Supplementary Materials

The following are available online at https://www.mdpi.com/article/10.3390/biomedicines9111689/s1. Table S1. PRISMA checklist. Table S2. Search strategies. Table S3. Articles included in the review. Table S4. Characteristics of the Systematic Reviews and Meta-Analyses analysed. Table S5. Summary of evidence. Effectiveness of the interventions analysed. Table S6. Assessing the quality of reviews using ROBIS tool (Tool to assess Risk of Bias in Systematic Reviews).
