# Supplementary material for: Efficacy of Acetylcholinesterase Inhibitors on Cognitive Function in Alzheimer’s Disease. Review of Reviews
Source: Biomedicines. 2021 Nov 15;9(11):1689. doi: 10.3390/biomedicines9111689 (PMC8615650; doi:10.3390/biomedicines9111689)
Supplement: Supplementary file 1 [file biomedicines-09-01689-s001.zip › biomedicines-1453112-supplementary.pdf]

Supplementary Materials:

Table S1. PRISMA checklist.

Section and Topic	Item #	Checklist item	Location where item is reported
**TITLE**			
Title	1	Identify the report as a systematic review.	1
**ABSTRACT**			
Abstract	2	See the PRISMA 2020 for Abstracts checklist.	1
**INTRODUCTION**			
Rationale	3	Describe the rationale for the review in the context of existing knowledge.	1-2
Objectives	4	Provide an explicit statement of the objective(s) or question(s) the review addresses.	2
**METHODS**			
Eligibility criteria	5	Specify the inclusion and exclusion criteria for the review and how studies were grouped for the syntheses.	3
Information sources	6	Specify all databases, registers, websites, organisations, reference lists and other sources searched or consulted to identify studies. Specify the date when each source was last searched or consulted.	3
Search strategy	7	Present the full search strategies for all databases, registers and websites, including any filters and limits used.	Table S2
Selection process	8	Specify the methods used to decide whether a study met the inclusion criteria of the review, including how many reviewers screened each record and each report retrieved, whether they worked independently, and if applicable, details of automation tools used in the process.	3
Data collection process	9	Specify the methods used to collect data from reports, including how many reviewers collected data from each report, whether they worked independently, any processes for obtaining or confirming data from study investigators, and if applicable, details of automation tools used in the process.	3
Data items	10a	List and define all outcomes for which data were sought. Specify whether all results that were compatible with each outcome domain in each study were sought (e.g. for all measures, time points, analyses), and if not, the methods used to decide which results to collect.	4
	10b	List and define all other variables for which data were sought (e.g. participant and intervention characteristics, funding sources). Describe any assumptions made about any missing or unclear information.	4
Study risk of bias assessment	11	Specify the methods used to assess risk of bias in the included studies, including details of the tool(s) used, how many reviewers assessed each study and whether they worked independently, and if applicable, details of automation tools used in the process.	4
Effect measures	12	Specify for each outcome the effect measure(s) (e.g. risk ratio, mean difference) used in the synthesis or presentation of results.	4
Synthesis methods	13a	Describe the processes used to decide which studies were eligible for each synthesis (e.g. tabulating the study intervention characteristics and comparing against the planned groups for each synthesis (item #5)).	4
	13b	Describe any methods required to prepare the data for presentation or synthesis, such as handling of missing summary statistics, or data conversions.	4
	13c	Describe any methods used to tabulate or visually display results of individual studies and syntheses.	4
	13d	Describe any methods used to synthesize results and provide a rationale for the choice(s). If meta-analysis was performed, describe the model(s), method(s) to identify the presence and extent of statistical heterogeneity, and software package(s) used.	NA

Section and Topic	Item #	Checklist item	Location where item is reported
	13e	Describe any methods used to explore possible causes of heterogeneity among study results (e.g. subgroup analysis, meta-regression).	NA
	13f	Describe any sensitivity analyses conducted to assess robustness of the synthesized results.	NA
Reporting bias assessment	14	Describe any methods used to assess risk of bias due to missing results in a synthesis (arising from reporting biases).	4
Certainty assessment	15	Describe any methods used to assess certainty (or confidence) in the body of evidence for an outcome.	NA
**RESULTS**			
Study selection	16a	Describe the results of the search and selection process, from the number of records identified in the search to the number of studies included in the review, ideally using a flow diagram.	4-5
	16b	Cite studies that might appear to meet the inclusion criteria, but which were excluded, and explain why they were excluded.	5 and Fig.1
Study characteristics	17	Cite each included study and present its characteristics.	5 and Table S4
Risk of bias in studies	18	Present assessments of risk of bias for each included study.	5 and Table S6
Results of individual studies	19	For all outcomes, present, for each study: (a) summary statistics for each group (where appropriate) and (b) an effect estimate and its precision (e.g. confidence/credible interval), ideally using structured tables or plots.	6 and Table S5
And Results of syntheses	20a	For each synthesis, briefly summarise the characteristics and risk of bias among contributing studies.	7 and Table S6
	20b	Present results of all statistical syntheses conducted. If meta-analysis was done, present for each the summary estimate and its precision (e.g. confidence/credible interval) and measures of statistical heterogeneity. If comparing groups, describe the direction of the effect.	Table S5
	20c	Present results of all investigations of possible causes of heterogeneity among study results.	Table S5
	20d	Present results of all sensitivity analyses conducted to assess the robustness of the synthesized results.	NA
Reporting biases	21	Present assessments of risk of bias due to missing results (arising from reporting biases) for each synthesis assessed.	Table S6
Certainty of evidence	22	Present assessments of certainty (or confidence) in the body of evidence for each outcome assessed.	7 and Table S6
**DISCUSSION**			
Discussion	23a	Provide a general interpretation of the results in the context of other evidence.	8-9
	23b	Discuss any limitations of the evidence included in the review.	10
	23c	Discuss any limitations of the review processes used.	10
	23d	Discuss implications of the results for practice, policy, and future research.	11
**OTHER INFORMATION**			
Registration and protocol	24a	Provide registration information for the review, including register name and registration number, or state that the review was not registered.	1-2
	24b	Indicate where the review protocol can be accessed, or state that a protocol was not prepared.	NA

Section and Topic	Item #	Checklist item	Location where item is reported
	24c	Describe and explain any amendments to information provided at registration or in the protocol.	NA
Support	25	Describe sources of financial or non-financial support for the review, and the role of the funders or sponsors in the review.	11
Competing interests	26	Declare any competing interests of review authors.	11
Availability of data, code and other materials	27	Report which of the following are publicly available and where they can be found: template data collection forms; data extracted from included studies; data used for all analyses; analytic code; any other materials used in the review.	17-37

NA (not applicable).

Table S2. Search strategies.

Electronic databases	Search
**PubMed**	(Alzheimer OR Alzheimer's disease) AND (cholinesterase inhibitor OR donepezil OR galantamine OR rivastigmine)<br><br>Limit: systematic review
**Cochrane Central Register of systematic reviews.**	(TS: Alzheimer OR Alzheimer's disease) AND (cholinesterase inhibitor OR donepezil OR galantamine OR rivastigmine) AND TI: systematic review
**Web of science y Ovid SP (Medline, Embase, PsycINFO, CINAHL y Lilacs etc…)**	(TS: Alzheimer OR Alzheimer's disease) AND (cholinesterase inhibitor OR donepezil OR galantamine OR rivastigmine) AND (TI, TS: systematic review)

Table S3. Articles included in the review.

Birks J. Cholinesterase inhibitors for Alzheimer's disease. The Cochrane database of systematic reviews. 2006(1):Cd005593.
Blanco-Silvente L, Castells X, Saez M, Barceló MA, Garre-Olmo J, Vilalta-Franch J, et al. Discontinuation, Efficacy, and Safety of Cholinesterase Inhibitors for Alzheimer's Disease: a Meta-Analysis and Meta-Regression of 43 Randomized Clinical Trials Enrolling 16 106 Patients. The international journal of neuropsychopharmacology. 2017;20(7):519-28.
Bond M, Rogers G, Peters J, Anderson R, Hoyle M, Miners A, et al. The effectiveness and cost-effectiveness of donepezil, galantamine, rivastigmine and memantine for the treatment of Alzheimer's disease (review of Technology Appraisal No. 111): a systematic review and economic model. Health technology assessment (Winchester, England). 2012;16(21):1-470.
Clegg A, Bryant J, Nicholson T, McIntyre L, De Broe S, Gerard K, et al. Clinical and cost-effectiveness of donepezil, rivastigmine, and galantamine for Alzheimer's disease. A systematic review. International journal of technology assessment in health care. 2002;18(3):497-507.
Grimmer T, Kurz A. Effects of cholinesterase inhibitors on behavioural disturbances in Alzheimer's disease - A systematic review. Drugs & Aging. 2006;23(12):957-67.
Hansen RA, Gartlehner G, Webb AP, Morgan LC, Moore CG, Jonas DE. Efficacy and safety of donepezil, galantamine, and rivastigmine for the treatment of Alzheimer's disease: a systematic review and meta-analysis. Clinical interventions in aging. 2008;3(2):211-25.
Hyde C, Peters J, Bond M, Rogers G, Hoyle M, Anderson R, et al. Evolution of the evidence on the effectiveness and cost-effectiveness of acetylcholinesterase inhibitors and memantine for Alzheimer's disease: systematic review and economic model. Age and ageing. 2013;42(1):14-20.
Kaduszkiewicz H, Zimmermann T, Beck-Bornholdt HP, van den Bussche H. Cholinesterase inhibitors for patients with Alzheimer's disease: systematic review of randomised clinical trials. BMJ (Clinical research ed). 2005;331(7512):321-7.

Table S3. (Continued) Articles included in the review.

Kobayashi H, Ohnishi T, Nakagawa R, Yoshizawa K. The comparative efficacy and safety of cholinesterase inhibitors in patients with mild-to-moderate Alzheimer's disease: a Bayesian network meta-analysis. International journal of geriatric psychiatry. 2016;31(8):892-904.
Livingston G, Katona C. How useful are cholinesterase inhibitors in the treatment of Alzheimer's disease? A number needed to treat analysis. International journal of geriatric psychiatry. 2000;15(3):203-7.
Ritchie CW, Ames D, Clayton T, Lai R. Metaanalysis of randomized trials of the efficacy and safety of donepezil, galantamine, and rivastigmine for the treatment of Alzheimer disease. American journal of geriatric psychiatry. 2004;12(4):358-69.
Takeda A, Loveman E, Clegg A, Kirby J, Picot J, Payne E, et al. A systematic review of the clinical effectiveness of donepezil, rivastigmine and galantamine on cognition, quality of life and adverse events in Alzheimer's disease. International journal of geriatric psychiatry. 2006;21(1):17-28.
Trinh NH, Hoblyn J, Mohanty S, Yaffe K. Efficacy of cholinesterase inhibitors in the treatment of neuropsychiatric symptoms and functional impairment in Alzheimer disease: a meta-analysis. Jama. 2003;289(2):210-6.

Table S4. Characteristics of the Systematic Reviews and Meta-Analyses analysed.

Articles	Type of Review (Review period)	Objectives and Questions	Database search	Inclusion/Exclusion Criteria	Studies (Participants)
**Birks, 2016 [11]**	Systematic review and meta-analysis. (1861 - 2005)	**P**: AD.<br>**I**: Donepezil, galantamine and rivastigmine.<br>**C**: placebo/AChEI.<br>**O**: ADAS-Cog MMSE, CIBIC-Plus and NPI.	CENTRAL, MEDLINE, EMBASE, PsycINFO, CINAHL, SIGLE, ISTP, INSIDE, ASLIB, DA, ADEAR, RENATI, CTc, AS, MRC, HTA NHS´s R+D, SANRA, DAV, NIHCs, clinicalTrials.gov, LILACS, PCDB, Copernic, Register CDCIG, FDA, EMEA, NICE and GCO.	Inclusion**:**<br>RCT<br>Double-blind<br>Placebo control<br>≥24 weeks<br>Mild to moderate or severe AD [a]<br>**Exclusion:**<br>No RCTs<br>No results and/or unclear	**13 RCTs (7298)**<br>*13 RCTs (AChEI/placebo):*<br>7 IG donepezil (2228)<br>5 IG galantamine (2267) y<br>3 IG rivastigmine (2803).
**Blanco-Silvente, L. et al. 2017 [18]**	Systematic review and meta-analysis. (1960 - 2016)	**P:** AD.<br>**I:** Donepezil, galantamine and rivastigmine.<br>**C:** Placebo/AChEI.<br>**O:** ADAS-Cog, MMSE, CIBIC-Plus and NPI.	MEDLINE CENTRAL, PsycINFO, Web of Knowledge, clinicaltrials.gov, clinicaltrialregister.eu, controlled-trials.com and PCDB.	**Inclusion:**<br>RCT<br>Placebo control<br>≥ 12 weeks<br>AD [a]<br>**Exclusion:**<br>Abstracts and/or patients with dementias non-AD.	**43 RCTs (16106)**<br>*43 RCTs (AChEI/placebo):*<br>23 IG donepezil (5755)<br>11 IG galantamine (6251) y<br>9 IG rivastigmine (4100).
**Bond, M. et al. 2012 [19]**	Systematic review and meta-analysis. Economic model. (2004 – 2010)	**P:** AD.<br>**I:** Donepezil, galantamine and rivastigmine.<br>**C:** Placebo/AChEI/ Memantine.<br>**O:** ADAS-Cog, MMSE, CIBIC-Plus and NPI.	Library Cochrane (CDSR y CENTRAL), MEDLINE, MEDLINE In-Process & Other Non-Indexed Citations, EMBASE, PsycINFO, EconLit, ISI Web of Science Databases - Science Citation Index, Conference Proceedings Citation Index and BIOSIS, CRD and clinicaltrials.gov.	**Inclusion**:<br>RCT and SR.<br>Placebo control/memantine/AChEI.<br>12-24 weeks<br>Evaluation of: [b]<br>**Exclusion:**<br>No RCTs (except CT con AE).<br>No results and/or unclear.	**17 RCTs (3965):**<br>*12 RCTs (AChEI/placebo):*<br>5 IG donepezil (234)<br>3 IG galantamine (1386) y<br>3 IG rivastigmine (1995).<br>*4 RCTs (AChEI/AChEI),*<br>*1 RCT (memantine/AChEI),*<br>**4 SRs**

Table S4. (Continued) Characteristics of the Systematic Reviews and Meta-Analyses analysed.

Articles	Type of Review (Review period)	Objectives and Questions	Database search	Inclusion/Exclusion Criteria	Studies (Participants)
**Clegg, A. et al. 2002 [20]**	Systematic review. (1960 – 2001)	**P:** AD.<br>**I:** Donepezil, galantamine and rivastigmine.<br>**C:** Placebo.<br>**O:** ADAS-Cog, MMSE and CIBIC-Plus.	MEDLINE, EMBASE, Library Cochrane, Database of Abstracts of Reviews of Effectiveness, NHS Economic Evaluation Database, National Research Register, Science Citation Index, BIOSIS, EconLit, MRC Trials database, Early Warning System, Current Controlled Trials, TOXLINE, Index of Scientific and Technical Proceedings, and Getting Easier Access to Reviews.	**Inclusion:**<br>RCT and EcE<br>Placebo control<br>AD<br>Cognition and cost-effectiveness [c].<br>**Exclusion:** Abstracts or conference presentations without peer review.	**15 RCTs (6753)**<br>*15 RCTs (AChEI/placebo):*<br>6 IG donepezil (2243)<br>4 IG galantamine (2520) y<br>5 IG rivastigmine (1990).
**Grimmer, T. et al. 2006 [21]**	Systematic review. (1960 – 2006)	**P**: AD<br>**I**: Donepezil, galantamine and tacrine.<br>**C**: Placebo.<br>**O**: ADAS-nonCog and NPI.	MEDLINE, EMBASE, Library Cochrane.	**Inclusion**:<br>RCT<br>Placebo control<br>AD [a]<br>**Exclusion: -**	**14 RCTs (4625)**<br>*14 RCTs (AChEI/placebo):*<br>6 IG donepezil (1598)<br>2 IG galantamine (1364) y<br>6 IG tacrine (1663).
**Hansen, R. A. et al. 2008 [22]**	Systematic review and meta-analysis. (1980 – 2007)	**P**: AD<br>**I**: Donepezil, galantamine and rivastigmine.<br>**C**: Placebo.<br>**O**: ADAS-Cog, CIBIC-Plus and NPI.	MEDLINE, EMBASE, Library Cochrane, CDER[c] and clinicaltrials.gov[d].	**Inclusion**:<br>$RCT_{(S)}$<br>Double blind<br>Placebo control/AChEI<br>≥ 12 weeks<br>Evaluation of: [b]<br>**Exclusion:** $RCT_{(S)}$ with significant statistical differences or "poor" quality for validity.	**27 RCTs (-)**<br>*23 RCTs (AChEI/placebo):*<br>12 IG donepezil (-)<br>8 IG galantamine (-) y<br>3 IG rivastigmine (-).<br>*4 RCTs (AChEI/AChEI):*<br>Donepez/galantam. (302)<br>Donepez/rivastigm (1105).
**Hyde, C. et al. 2013 [23]**	Systematic review and meta-analysis. Economic model. (2004 – 2010)	**P:** AD<br>**I:** Donepezil, galantamine and rivastigmine.<br>**C:** Placebo.<br>**O:** ADAS-Cog, CIBIC-Plus and NPI.	MEDLINE, MEDLINE In Process, EMBASE, Library Cochrane (CDSR y CENTRAL), PsycINFO, EconLIT, ISI Web of Science Databases: Science Citation Index, Conference Proceedings Citation Index, BIOSIS, CRD, clincaltrials.gov and BDCF.	**Inclusion**:<br>$RCT_{(S)}$<br>Placebo control<br>≥ 12-24 weeks<br>Mild to moderate AD [a]<br>Evaluation of: [e]<br>**Exclusion:** Studies without inclusion criteria.	**14 RCTs (6716)**<br>*14 RCTs (AChEI/placebo):*<br>5 IG donepezil (1414)<br>5 IG galantamine (2884) y<br>4 IG rivastigmine (2418).

Table S4. (Continued) Characteristics of the Systematic Reviews and Meta-Analyses analysed.

Articles	Type of Review (Review period)	Objectives and Questions	Database search	Inclusion/Exclusion Criteria	Studies (Participants)
**Kaduszkiewicz. H. et al. 2005 [24]**	Systematic review. (1989 – 2004)	**P:** AD<br>**I:** Donepezil, galantamine and rivastigmine.<br>**C:** Placebo.<br>**O:** ADAS-Cog, CIBIC-Plus and NPI.	MEDLINE, EMBASE, Library Cochrane (CDSR).	**Inclusion**:<br>RCT<br>Double-blind<br>Placebo control<br>≥12 weeks<br>AD probable or possible [a]<br>**Exclusion:** CT no clinical outcomes or VD. No direct comparisons between AChEI.	**22 RCTs (8970)**<br>22 *RCTs (AChEI/placebo):*<br>12 IG donepezil (2354)<br>5 IG galantamine (2012) y<br>5 IG rivastigmine (1329).
**Kobayashi, H. et al. 2016 [25]**	Systematic review and meta-analysis. (1960 – 2014)	**P:** Mild to moderate AD<br>**I:** Donepezil, galantamine and rivastigmine.<br>**C:** Placebo.<br>**O:** ADAS-Cog. CIBIC-Plus and NPI.	MEDLINE, EMBASE, Library Cochrane and manually the cognitive enhancement group.	**Inclusion**:<br>RCT<br>Double-blind<br>Placebo control/AChEI<br>Mild to moderate AD<br>Evaluation of: [b]<br>**Exclusion:** AD severe [a] o MCI and $RCT_{(S)}$ [f]	**21 RCTs (9509)**<br>18 *RCTs (AChEI/placebo):*<br>10 IG donepezil (-)<br>6 IG galantamine (-) y<br>9 IG rivastigmine (-).<br>*3 RCTs (AChEI/AChEI)*
**Livingston, G. et al. 2000 [26]**	Systematic review. (1966 – 1999)	**P:** AD.<br>**I:** Donepezil, rivastigmine and tacrine.<br>**C:** Placebo.<br>**O:** ADAS-Cog, MMSE and CIBIC-Plus.	MEDLINE, EMBASE, Psychlit.	**Inclusion**:<br>RCT (≥10 patients con AD).<br>Double-blind<br>≥12 weeks<br>Evaluation of: [g]<br>**Exclusion:** $RCT_{(S)}$ crossover or open-label.	**5 RCTs (1312)**<br>5 *RCTs (AChEI/placebo):*<br>1 IG donepezil (152)<br>2 IG rivastigmine (391) y<br>1 IG tacrine (64).
**Ritchie, C. W. et al. 2004 [27]**	Systematic review and meta-analysis. (1992 – 2002)	**P:** AD.<br>**I:** Donepezil, galantamine and rivastigmine<br>**C:** Placebo.<br>**O:** ADAS-Cog, MMSE and CIBIC-Plus.	MEDLINE, EMBASE, PsycINFO and Library Cochrane (CENTRAL), International Pharmaceutical Abstracts, SIGLE, ASLIB and Summaries of Oral Presentations and the Register of Current Control Trials of the Medical Research Council. PCDB.	**Inclusion**:<br>$RCT_{(S)}$ (ITT) y SRMA (Cochrane)<br>Double-blind<br>Placebo control<br>AD [a].<br>**Exclusion:** $RCT_{(S)}$ double-blind [h]	**21 RCTs (7701)**<br>5 *RCTs (AChEI/placebo):*<br>9 IG donepezil (3321)<br>6 IG galantamine (3390) y<br>15 IG rivastigmine. (1990).

Table S4. (Continued) Characteristics of the Systematic Reviews and Meta-Analyses analysed.

Articles	Type of Review (Review period)	Objectives and Questions	Database search	Inclusion/Exclusion Criteria	Studies (Participants)
**Takeda, A. et al.2006 [28]**	Systematic review. (1960 – 2004)	**P:** Mild to moderate AD<br>**I:** Donepezil, galantamine and rivastigmine.<br>**C:** Placebo.<br>**O:** ADAS-Cog, MMSE and NPI.	MEDLINE, EMBASE, Library Cochrane, PsychInfo and eight other electronic databases.	**Inclusion**:<br>RCT of AChEI,<br>Placebo control/AChEI<br>Mild to moderate AD [i]<br>Assessment of cognition using the ADAS-Cog<br>**Exclusion:** RCT(S) where the main dementia was not AD.	**26 RCTs (9856)**<br>23 *RCTs (AChEI/placebo):*<br>13 IG donepezil (2466)<br>6 IG galantamine (2329) y<br>4 IG rivastigmine. (1375).<br>*3 RCTs (AChEI/AChEI).*
**Trinh N. et al.2003 [29]**	Systematic review and meta-analysis. (1966 – 2001)	**P:** Mild to moderate AD<br>**I:** Donepezil, galantamine and tacrine.<br>**C:** Placebo.<br>**O:** ADAS-nonCog or NPI.	MEDLINE, Library Cochrane (CENTRAL), PsychInfo, BIOSIS, PubMed, Dissertations Abstracts On-line and PCDB.	**Inclusion**:<br>RCT<br>Double-blind parallel or crossover<br>Placebo control<br>Mild to moderate AD [a]<br>Evaluation of: [j]<br>**Exclusion:** Studies combining functional measures of ADL and IADL domains.	**8 RCTs (3270)**<br>1 IG Donepezil (286)<br>2 IG galantamine (1364) y<br>5 IG tacrine (1620).

P, Patients; I, Intervention; C, Comparator; O, Outcome; AD, Alzheimer's disease; AChEI, Acetylcholinesterase inhibitors; RCT(S), Randomised clinical trial(S); -, Without data; ADAS-Cog, *Alzheimer´s Disease Assessment Scale, cognitive subscale; ADAS-nonCog, Alzheimer´s Disease Assessment Scale, non-cognitive subscale;* MMSE, *Mini-Mental State Examination*; CIBIC-Plus, *Clinician´s Interviewed-Based Impression of Change Scale*; NPI, *Neuropsychiatric instrument*; CENTRAL, Cochrane Central Register of Controlled Trials; CDSR, Cochrane Systematic Reviews Register; SIGLE, Grey Literature in Europe; ISTP, Index of Scientific and Technical Proceedings; INSIDE, Proceedings and Conference Database; ASLIB, Aslib Index of UK and Irish Theses; DA, Dissertation Abstract from USA; ADEAR, AD Clinical Trials Database; RENATI, UK and Irish National Research Register; CTc, Current Clinical Trials; CT, Clinical Trial; AS, Alzheimer's Society; MRC, Medical Research Council of Hong Kong Health Services Research Fund; HTA NHS´s R+D, Health Technology Assessment NHS´s research and developed; NHS, UK National Health Service; SANRA, South Australian Network for Research on Ageing; DVA, US Department of Veterans Affairs; NIHCs, National Institutes of Health Cooperative Studies; LILACS, Latin American and Caribbean Health Sciences Literature; PCDB, Pharmaceutical Companies Database; FDA, U.S. Food and Drug Administration; EMEA, Europe. Middle East and Africa; NICE, The National Institute for Health and Care Excellence; GCO, Global Clinical Operations; CIE-10, Tenth Revision of the International Classification of Diseases; DSM, Diagnostic and Statistical Manual of Mental Disorders; NINCDS-ADRDA, National Institute of Neurological and Communicative Diseases and Stroke-Alzheimer's Diseases clinical relevance; IG, Intervention group; EMA, European Medicines Agency; MDET, Randomised placebo-controlled clinical trials; CRD, The Centre for Reviews and Dissemination databases HTA, DARE and NHS EDD. SR, Systematic review**;** AE, adverse event; EcE, Economic Studies; CDER, US Centre for Drug Evaluation and Research.; VD, Vascular Dementia; MCI, Mild cognitive impairment**;** ITT, Intention to treat; SRMA, Systematic reviews with meta-analysis).

[a] Probable or diagnosed according to ICD-10 [30], DSM-V [31], and NINCDS-ADRDA criteria [32].

[b] Cognition, function, behaviour and/or global clinical assessment of change.

Table S4. (Continued) Characteristics of the Systematic Reviews and Meta-Analyses analysed.

[c] Incremental cost/year spent.

[d] Manual searches.

[e] Severity of illness, response to treatment, behavioural symptoms, mortality, ability to remain independent, likelihood of admission to residential/nursing care, health-related quality of life of patients and caregivers, adverse effects of treatment, efficacy and costs.

[f] At high or low doses of AChEI.

[g] Cognition, general assessment or activities of daily living.

[h] Open-label, single-arm (no AChEI or AD patients) or not fully published and did not use NINCDS-ARDA inclusion criteria for AD.

[i] Diagnosed with NINCDS-ADRDA or DSM-III/IV criteria [32].

[j] Behaviour with ADAS-nonCog or NPI and functional outcomes measured by separate ADL and IADL domain scores.

Table S5. Summary of evidence. Effectiveness of the interventions analysed.

Articles	Outcome measure	Results	Conclusions	Risk of bias assessment	Meta-analysis methods	Meta-analysis results	Publ. bias
**Birks, 2016 [11]**	SMD, OR (95% CI)	The results of 10 double-blind, placebo-controlled RCTs demonstrate that treatment for 6 months with **donepezil, galantamine or rivastigmine** at the recommended dose for people with mild, moderate or severe dementia due to AD resulted in improvements in cognitive function, on average: [-2.37 points (-2.73 to -2.02); *p* < 0.01]. The effects are similar for patients with severe dementia, although there is very little evidence. There are also benefits associated with AChEI compared to placebo after approximately 6 months of treatment with respect to global change and behavioural disturbances.	All three AChEI are effective in mild to moderate AD. Despite slight variations in the mode of action of the three AChEI, there is no evidence of differences between them with respect to efficacy. Evidence from one large trial shows fewer adverse events associated with donepezil compared to rivastigmine.	CG of Cochrane [14]	Fixed effects model [a].	**Cognitive** assessment, ADAS-Cog[b]:<br>[n=10; N= 4236; SMD= -2.37 (-2.73 a -2.02); *p* = < 0.01].<br><br>**Cognitive** assessment, MMSE[b]:<br>[n= 9; N= 3118; SMD= 1.37 (1.13 a 1.61); *p* = < 0.01].<br><br>**Global clinical** assessment, CIBIC-Plus[b]:<br>[n= 8; N= 3402; OR= 1.56 (1.32 a 1.85); *p* < 0.01].<br><br>**Behavioural** assessment NPI-TOTAL[b]:<br>[n= 3; N= 1005; DME = -2.44 (-4.12 a -0.76); *p* < 0.01].	NA
**Blanco-Silvente, L. et al. 2017 [18]**	SMD, OR (95% CI)	**AChEI** improved cognitive function (SMD = 0.38) and global symptomatology (SMD = 0.28) but not neuropsychiatric symptoms. **Donepezil** showed greater efficacy in global change (SMD = 0.41).<br>Mortality was lower in IAChE than placebo (OR = 0.65).	AChEI show a poor risk-benefit ratio, as indicated by mild symptom improvement and higher all-cause discontinuation than placebo, a reduction in mortality was suggested.<br>Intervention-related and patient-related factors modify the effect of cholinesterase inhibitors in patients with AD.	CG of Cochrane [14]	Meta regression with Bayesian framework.	**Cognitive** assessment, ADAS-Cog o MMSE:<br>[n=41, SMD= 0.38 (0.28 a 0.47); I_2(%) = 41.1].<br><br>**Global clinical** assessment, CIBIC-Plus:<br>[n=32; SMD= 0.28 (0.22 a 0.34); I_2(%) = 0].<br><br>**Behavioural** assessment, NPI:<br>[n= 19; SMD= 0.03 (-0.04 a 0.09); *p* < 0.01].<br><br>Not statistically significant effects in multivariate analysis. No covariate had a statistically significant effect on discontinuation due to lack of efficacy (on cognitive function, functional capacity, proportion of patients with AD and mortality).	Funnel plots [36].

Table S5. (Continued) Summary of evidence. Effectiveness of the interventions analysed.

Articles	Outcome measure	Results	Conclusions	Risk of bias assessment	Meta-analysis methods	Meta-analysis results	Publ. bias
**Bond, M. et al. 2012 [19]**	WMD, SMD (95% CI)	**Donepezil** has a dose-related beneficial effect at 10mg/ml. Confidence on the size and statistical significance of the effect estimates of **galantamine, rivastigmine and memantine** improved function and overall impact in particular.	The additional evidence of clinical efficacy identified in this SR update continues to suggest a clinical benefit of AChEI in alleviating AD symptoms, although there is considerable debate about the magnitude of the effect. There is also some evidence that AChEI have an impact on the control of disease progression. Although there is also new evidence on the efficacy of memantine, it remains less favourable to the use of memantine than the evidence for AChEI.<br>.	Modified NHS quality criteria [35], CRD-V [32]	Random effects model. Aggregate data [c].	For **donepezil** [d]**:**<br>**Cognitive** assessment, ADAS-Cog:<br>[WMD = -2.90 (-3.61 a -2.18)]; $p < 0.01$];<br>**Cognitive** assessment, MMSE:<br>[WMD = 1.21 (0.84 a 1.57); $p < 0.01$];<br>**Global clinical** assessment, CIBIC-Plus:<br>[WMD = -0.43 (-0.55 a -0.31); $p < 0.01$].<br>For **galantamine** [e]**:**<br>**Cognitive** assessment, ADAS-Cog: [WMD = -2.96 (-3.41 a -2.51); $p < 0.01$];<br>**Global clinical** assessment, CIBIC-Plus: [WMD = -0.20 (-0.30 -0.09); $p<0.01$] (≤24mg/day [f])<br>**Behavioural** assessment, NPI: [WMD = -1.46 (-2.59, -0.34); $p = 0.012$].<br>For **rivastigmine** [g]:<br>**Cognitive** assessment, ADAS-Cog: [WMD = -2.46 (-3.37, -1.56); $p < 0.01$];<br>**Cognitive** assessment, MMSE: [WMD = 1.02 (0.63 a 1.41); p < 0.01];<br>**Global clinical** assessment, CIBIC-Plus: [WMD = -0.42 (-0.55 a -0.29); $p < 0.01$];<br>**Behavioural** assessment, NPI: Mixed results (could not be grouped due to high heterogeneity).<br>**Efficacy:** 9.5 mg/day patch = 12.5 mg/day capsule, but with less AE.<br><br>*4 RCTs (AChEI/AChEI),*<br>**Cognitive** assessment [h]**: Donepezil** was most effective at short-term follow-up using ADAS-cog and MMSE, and remained so for MMSE at 24-26 weeks; for **galantamine** ADAS-cog was more favourable.<br>**Behavioural** assessment: **donepezil** was more favourable.<br>**Global clinical** assessment: results less clear, with **galantamine** being better treatment at 12-16 weeks as measured by CIBIC-plus, but **donepezil** prevailed at 24-28 weeks.<br>*1 RCT (memantine/AChEI),*<br>**Combination (MTC** [i]), **(memantine + AChEI):** No benefit in cognitive, behavioural or global outcomes.	Funnel plots [36].

Table S5. (Continued) Summary of evidence. Effectiveness of the interventions analysed.

Articles	Outcome measure	Results	Conclusions	Risk of bias assessment	Meta-analysis methods	Meta-analysis results	Publ. bias
**Clegg, A. et al. 2002 [20]**	NA	12 of the 15 included RCTs were of good quality and only two of poor quality. Although only **donepezil** and **galantamine** had beneficial effects on global cognition these improvements were small and may not be clinically meaningful. Behavioural measures were rarely assessed.	Donepezil, rivastigmine and galantamine appear to have some clinical effect in people with AD, although it is unclear to what extent they translate into real differences in daily life.	Jadad Scale [34]	NA	NA	NA
**Grimmer, T. et al. 2006 [21]**	NA	A statistically significant difference between active treatment and placebo on behavioural symptoms was observed in three of the 14 studies. Treatment effects ranged from 2.0 to 6.2 points on the NPI.<br>Pooled analysis showed a small but statistically significant advantage over placebo on the NPI scale and a trend towards benefit on the ADAS-nonCog scale.	AChEI have modest effects when used as a general treatment for the AD group of behavioural disturbances. With regard to the limitations of the available trials, and in view of the risks associated with the use of atypical antipsychotics, the potential of AChEI for the management of specific neuropsychiatric symptoms in AD patients should be explored in further studies.	NA	NA	NA	NA
**Hansen, R. A. et al. 2008 [22]**	WMD (ADAS-Cog and NPI), RR (95% CI) (CIBIC-Plus).	MAs of placebo-controlled data support the modest overall benefits of the drugs in stabilising or slowing decline in cognition, function, behaviour and overall clinical change. Three open-label trials and one randomised double-blind trial directly compared donepezil with galantamine and rivastigmine. The results are contradictory; two studies suggest that there is no difference in efficacy between the drugs compared, while one study found donepezil more effective than galantamine, and one study found rivastigmine more effective than donepezil.	All three AChEI equally, versus placebo are shown to be more effective in stabilising or slowing deterioration in cognition, behaviour and global change (in adjusted indirect comparisons suggesting that **donepezil** and **rivastigmine** may be slightly more effective than galantamine. Lower incidence of common AE for donepezil and higher with rivastigmine.<br>Further high-quality comparative evidence is needed to confirm these conclusions.	Modified NHS quality criteria [35], CRD-V [32]	Random effects model. Aggregate data.	14 RCTs **Cognitive** assessment, ADAS-Cog:<br>[n donepezil= 5; = WMD -2.67 (-3.28 a -2.06); I_2= 0%];<br>[n galantamine= 7; WMD= -2.76 (-3.17 a -2.34); I_2= 0%];<br>[n rivastigmine= 2; WMD= -3.01 (-3.80 a -2.21); I_2= 70%].<br><br>9 RCTs **Global clinical** assessment, CIBIC-Plus:<br>[n donepezil= 3; RR= 1.88 (1.5 a 2.34); I_2= 0%];<br>[n galantamine= 4; RR= 1.15 (0.96 a 1.39); I_2= 0%];<br>[n rivastigmine= 2; RR= 1.64 (1.29 a 2.09); I_2= 0%].<br><br>7 RCTs **Behavioural** assessment, NPI:<br>[n donepezil= 4; WMD= -4.3 (-5.95 a -2.65); I_2= 43%];<br>[n galantamine= 3; WMD= -1.44 (-2.39 a -0.48); I_2= 0%];<br><br>The pooled estimates did not differ significantly when analyses were stratified by dose.	Funnel plots [36].

Table S5. (Continued) Summary of evidence. Effectiveness of the interventions analysed.

Articles	Outcome measure	Results	Conclusions	Risk of bias assessment	Meta-analysis methods	Meta-analysis results	Publ. bias
**Hyde, C. et al. 2013 [23]**	WMD (95% CI)	Confidence on the size and statistical significance of the effect estimates of **galantamine** and **rivastigmine** improved cognitive function and overall impact in particular.	The additional evidence of clinical efficacy identified in this SR update continues to suggest a clinical benefit of AChEI in alleviating AD symptoms, although there is considerable debate about the magnitude of the effect.<br>There has been a change in the evidence base between 2004 and 2010, in line with the change in NICE guideline guidance. It is possible that cost-effectiveness estimates may increase if there are changes in drug costs.	NA	Random effects model. Aggregate data.	11 RCTs **Cognitive** assessment**,** ADAS-Cog:<br>[n donepezil= 2; N= 850; WMD= -2.90 (-3.61 a -2.18)];<br>[n galantamine= 5; N= 2884; WMD= -2.96 (-3.41 a -2.51)] y<br>[n rivastigmine= 4; N= 2418; WMD= -2.46 (-3.37 a -1.56)].<br><br>9 RCTs **Global clinical** assessment, CIBIC-Plus:<br>[n donepezil= 3; N= 1051; WMD= -0.43 (-0.55 a -0.31)];<br>[n galantamine= 3; N= 1711; WMD= -0.20 (-0.30 a -0.09)] y<br>[n rivastigmine= 3; N= 1574; WMD= -0.42 (-0.55 a -0.29)].<br><br>5 RCTs **Behavioural** assessment, NPI:<br>[n donepezil= 2; N= 635; DMP= -3.12 (-8.2 a 1.9)];<br>[n galantamine= 2; N= 1644; DMP= -1.46 (-2.59 a -0.32)] y<br>[n rivastigmine= 1; N= 1195; No ≠ significant differences between rivastigmine and placebo].	NA
**Kaduszkiewicz. H. et al. 2005 [24]**	SMD (95% CI)	14 RCTs **cognition** assessment, ADAS-Cog:<br>[n = 12; SMD (1.5 and 3.9); $p < 0.05$ in favour of AChEI].<br><br>12 RCTs **global clinical** assessment, CIBIC-Plus:<br>[n =5; SMD (2.26 and 0.54); $p < 0.05$ in favour AChEI].<br>[n =5; (comparison proportions vs scale benefit); n=5 not ≠ significant].<br><br>2 RCTs **behaviour** assessment, NPI:<br>[n=2, not ≠ significant AChEI vs placebo].<br>AE reached 5% significance.	The flawed methods, small clinical benefits and scientific basis for AChEI recommendations for the treatment of AD is questionable.	NA [h]	NA	NA	NA

Table S5. (Continued) Summary of evidence. Effectiveness of the interventions analysed.

Articles	Outcome measure	Results	Conclusions	Risk of bias assessment	Meta-analysis methods	Meta-analysis results	Publ. bias
**Kobayashi, H. et al. 2016 [25]**	SDM, OR (95% CI), I_2	Among the 21 included trials, the network MA showed that **all** treatments were significantly **more effective** than placebo on **cognition** as measured by the ADAS-Cog. All treatments except galantamine were significantly more effective than placebo on global change as measured by the CIBIC-Plus.<br>No improvement was observed with respect to neuropsychiatric (behavioural) symptoms under AChEI treatment, as measured by the NPI.	First attempt to incorporate available direct and indirect evidence. Results suggest that AChEI should have significant efficacy for cognition and assessment of global change, but efficacy on neuropsychiatric symptoms is questionable in patients with mild to moderate AD.	NA	MA in sequential network [i].	All AChEI had an improvement in **cognition** (p= 0.606), significantly greater than placebo (p= 0.017), and the derived hierarchy was *galantamine> rivastigmine> donepezil.*<br><br>All AChEI had an improvement in **global clinical change** (p= 0.950), significantly greater than placebo (p= 0.044), and the derived hierarchy was *donepezil> rivastigmine> galantamine.*<br><br>AChEI had no significantly greater **behavioural** improvement than placebo, and the derived hierarchy was *galantamine> donepezil > rivastigmine.*	Funnel plots [36].
**Livingston, G. et al. 2000 [26]**	NNT, ARR, RR, RRR,	A small number of patients (in most cases between 3 and 7) need to be treated with appropriate doses of AChEI to improve clinical symptoms or postpone deterioration in one of them.	These small NNT suggest that, despite their cost, AChEI have a valuable place in the current clinical management of AD [j].	NA	NA	NA	NA
**Ritchie, C. W. et al. 2004[27]**	OR (95% CI)	All three drugs showed beneficial effects on cognitive tests compared to placebo.<br>For donepezil and rivastigmine, higher doses were associated with a greater effect. This was not the case for galantamine. The odds of overall clinical improvement demonstrated superiority over placebo for each drug, with no dose effects observed.<br>Dropout rates were higher with galantamine and rivastigmine. There was little difference in the dropout rate for each drug at each dose level, except for high-dose donepezil.<br>This was due to the high dropout rate in two 52-week studies using higher doses.	All three drugs have similar cognitive efficacy, with **donepezil** and **rivastigmine** showing a dose effect at all dosage levels studied. However, both galantamine and rivastigmine are associated with a higher risk of trial dropout than placebo, especially at higher dosing levels.	NA	Fixed effects model [k]	**Cognition** assessment, ADAS-Cog:<br>[n $\text{donepezil}_{5mg\text{-}10mg}$= 5; MD= -0.88 (-1.53 to -0.23); $F_{[1, 2.098]} = 7.04$; $p < 0.01$]<br>[n galantamine= 6; ($F_{[2, 3,381]} = 0.38$; $p = 0.68$)]; and<br>[n $\text{rivastigmine}_{6\text{-}12\text{ mg and }1\text{-}4\text{ mg}}$ = 4; MD= -2.06 (-2.94 to -1.18); $F_{[1, 1,420]} = 21.11$; $p < 0.01$].<br><br>**Global clinical** assessment, CIBIC-Plus:<br>[n donepezil= 4; χ^2= 0.17; p= 0.41] and [n donepezil= 5; χ^2= 0.50; p= 0.78] little dose-derived effect.<br>[n $\text{rivastigmine}_{\text{low dose}}$ = 4; OR= 1.68 (1.10 to 2.58); n $\text{rivastigmine}_{\text{high dose}}$ OR= 2.30 (1.51 to 3.52)] in favour of treatment.	NA

Table S5. (Continued) Summary of evidence. Effectiveness of the interventions analysed.

Articles	Outcome measure	Results	Conclusions	Risk of bias assessment	Meta-analysis methods	Meta-analysis results	Publ. bias
**Takeda, A. et al.2006 [28]**	WMD, *p* value	Treatment with AChEI resulted in significantly better cognitive performance using the ADAS-Cog scale compared to placebo. Overall, these findings were supported by the MMSE scale. The results of direct comparisons were limited by the small number of studies and the quality of the studies; overall, they do not show strong support for any drug.	AChEI, **donepezil, galantamine** and **rivastigmine** can delay cognitive decline in patients with mild to moderately severe AD for at least 6 months duration [j].	Modified NHS quality criteria [35], CRD-V [32]	NA	NA	NA
**Trinh N. et al.2003 [29]**	SMD, WMD (95% CI)	Patients randomised to AChEI improved 0.03 points on the ADAS-nonCog (0.00 to 0.05 points) and 1.72 points on the NPI (0.87 to 2.57 points). There was no difference in efficacy between various AChEI.	AChEI have beneficial impact on the neuropsychiatric and functional outcomes of patients with AD. Future research should focus on how such improvements translate into long-term outcomes such as patient quality of life, institutionalisation and caregiver burden.	NA	Random effects model.	**Behavioural** assessment:<br>ADAS- nonCog: [n=5; N= 1620; IG improvement 0.03 points vs placebo (0.00 to 0.05 points)].<br>NPI: [n=3; N= 1650; GI improves 1.72 points vs placebo (0.87 to 2.57 points)].<br>Indication of trend towards benefit in neuropsychiatric dysfunction from the use of AChEI. For both ADAS-nonCog MA and NPI, tests of heterogeneity were not statistically significant (*p*= 0.99 for both).	Funnel plots [36] and Kendall's Tau.

SMD, Standardised Mean Difference; WMD, Weighted Mean Difference; OR, Odds Ratio; RR, Relative Risk; 95% CI, 95% Confidence Interval; RCTs, Randomised Clinical Trials; AD, Alzheimer's Disease; AChEI, Acetylcholinesterase Inhibitors; CG, Collaborative Guideline; MA, Meta-Analysis; ITT, Intention to Treat; NNT, Number needed to treat; LOCF, Last Observation Carried Forward; ADAS-Cog, Alzheimer's Disease Assessment Scale, cognitive subscale; ADAS-nonCog, Alzheimer's Disease Assessment Scale, non-cognitive subscale; MMSE, Mini-Mental State Examination; CIBIC-Plus, Clinician's Interviewed-Based Impression of Change Scale; NPI, Neuropsychiatric instrument; n, number of RCTs; N, Number of participants; *p*, *p*-value; Publ., Publication; NA, Not applicable; mg, milligrams; ml, millilitres; SR, Systematic review; CRD, Center for Reviews and Dissemination; AE, Adverse event; MTC, Mixed-treatment comparisons: indirect comparisons; ≠, difference; *vs*, *versus*, in comparison with; I_2, Heterogeneity; ARR, Absolute relative risk; RRR, Risk reduction; IG, Intervention group.

[a] From the ITT population with incorporation of LOCF assessments.

[b] Mean changes in score from baseline to 6 months or more (ITT-LOCF).

[c] Analysis due to intrinsic clinical heterogeneity. Distribution χ^2 and the I_2 statistic.

[d] Pooled cognitive and general assessment outcomes showed a significant benefit of donepezil as measured by the ADAS-Cog, MMSE and CIBIC-Plus respectively, at 24-26 weeks of follow-up.

[e] Pooled cognitive, behavioural and general assessment outcomes also showed significant benefit from galantamine as measured by the ADAS-Cog, NPI and CIBIC-Plus respectively, at 21-26 weeks follow-up.

[f] At daily doses of

[g] Pooled cognitive and general assessment outcomes showed a significant benefit of rivastigmine as measured by ADAS-Cog, MMS and CIBIC-Plus respectively, at 24-26 weeks follow-up (≥12mg/day).

[h] Varied according to follow-up time and measurement used.

[i] Results of mixed-treatment comparisons: indirect comparisons.

[j] Criteria developed by the USA Preventive Services Task Force and the National Health Service Center for Reviews and Dissemination.

[h] Each study was assessed independently, following a pre-determined checklist of methodological quality criteria. It does not specify anything else.

[i] For efficacy and safety outcomes based on drug/dose for the treatment conditions.

[j] Copyright # 2000 John Wiley & Sons, Ltd.

[k] For each drug and dose level. Standard heterogeneity tests for MA were used.

Table S6. Assessing the quality of reviews using ROBIS tool (Tool to assess risk of bias in systematic reviews).

	**Birks, JS. 2016 [11]**	**Blanco-Silvente, L. et al. 2017[17] [18]**	**Bond, M. et al. 2012 [19]**	**Clegg, A. et al. 2002 [20]**	**Grimmer, T. et al. 2006 [21]**	**Hansen, R. A. et al. 2008 [22]**	**Hyde, C. et al. 2013 [23]**	**Kaduszkiewicz. H. et al. 2005 [24]**	**Kobayashi, H. et al. 2016 [25]**	**Livingston, G. et al. 2000 [26]**	**Ritchie, C. W. et al. 2004 [27]**	**Takeda, A. et al.2006 [28]**	**Trinh N. et al.2003 [29]**
**Phase 1. Assessing relevance. Review of interventions.**													
**Category**													
Patient/Population(s):	Alzheimer´s	Alzheimer´s	Alzheimer's	Alzheimer's	Alzheimer's	Alzheimer's	Alzheimer's	Alzheimer's	Alzheimer's	Alzheimer's	Alzheimer's	Alzheimer's	Alzheimer's
Intervention(s):	Donepezil, galantamine and rivastigmine	Donepezil, galantamine and rivastigmine	Donepezil, galantamine and rivastigmine	Donepezil, galantamine and rivastigmine	Donepezil, rivastigmine and tacrine	Donepezil, galantamine and rivastigmine	Donepezil, galantamine and rivastigmine	Donepezil, galantamine and rivastigmine	Donepezil, galantamine and rivastigmine	galantamin e and rivastigmin e	Donepezil, galantamine and rivastigmine	Donepezil, galantamine and rivastigmine	Donepezil, galantamine and tacrine
Comparator(s):	Versus placebo	Versus placebo	Versus placebo	Versus placebo	Versus placebo	Versus placebo	Versus placebo	Versus placebo	Versus placebo	Versus placebo	Versus placebo	Versus placebo	Versus placebo
Outcome(s):	ADAS-Cog., MMSE, CIBIS-Plus, NPI	ADAS-Cog., MMSE, CIBIS-Plus, NPI	ADAS-Cog., MMSE, CIBIS-Plus, NPI	ADAS-Cog., MMSE, CIBIS-Plus	ADAS-*NonCog*., NPI	ADAS-Cog., CIBIS-Plus, NPI	ADAS-Cog., CIBIS-Plus, NPI	ADAS-Cog., CIBIS-Plus, NPI	ADAS-Cog., CIBIS-Plus, NPI	ADAS-Cog., MMSE, CIBIS-Plus	ADAS-Cog., MMSE, CIBIS-Plus	ADAS-Cog., MMSE, NPI	ADAS-*NonCog*., NPI
Does the question addressed by the review match the target question?	☺	☺	☺	☺	☺	☺	☺	☺	☺	☺	☺	☺	☺
**Phase 2. Identifying concerns with the review process**													
**DOMAIN 1: STUDY ELIGIBILITY CRITERIA**													
Describe the study eligibility criteria, any restrictions on eligibility and whether there was evidence that objectives and eligibility criteria were pre-specified:													
1.1 Did the review adhere to pre-defined objectives and eligibility criteria?	Y	Y	Y	Y	Y	Y	Y	PY	Y	Y	Y	Y	Y
1.2 Were the eligibility criteria appropriate for the review question?	Y	Y	Y	PY	Y	Y	Y	PY	Y	Y	Y	Y	Y
1.3 Were the eligibility criteria unambiguous?	Y	PY	Y	PY	Y	Y	Y	PY	Y	Y	Y	Y	Y
1.4 Were all restrictions in eligibility criteria based on study characteristics appropriate (e.g. date, sample size, study quality, outcomes measured)?	Y	PY	Y	PY	Y	Y	Y	Y	Y	Y	Y	Y	Y
1.5 Were any restrictions in eligibility criteria based on sources of information appropriate (e.g. publication status or format, language, availability of data)?	Y	PY	Y	PY	Y	PY	Y	PY	Y	PY	Y	Y	Y
Concerns regarding specification of study eligibility criteria. Rationale for concern:	☺	☺	☺	☺	☺	☺	☺	☺	☺	☺	☺	☺	☺

Table S6. (Continued) Assessing the quality of reviews using ROBIS tool (Tool to assess risk of bias in systematic reviews).

DOMAIN 2: IDENTIFICATION AND SELECTION OF STUDIES	Birks, JS. 2016 [11]	Blanco-Silvente, L. et al. 2017 [18]	Bond, M. et al. 2012 [19]	Clegg, A. et al. 2002 [20]	Grimmer, T. et al. 2006 [21]	Hansen, R. A. et al. 2008 [22]	Hyde, C. et al. 2013 [23]	Kaduszkiewicz. H. et al. 2005 [24]	Kobayashi, H. et al. 2016 [25]	Livingston, G. et al. 2000 [26]	Ritchie, C. W. et al. 2004 [27]	Takeda, A. et al.2006 [28]	Trinh N. et al.2003 [29]
Describe methods of study identification and selection (e.g. number of reviewers involved):													
2.1 Did the search include an appropriate range of databases/electronic sources for published and unpublished reports?	Y	Y	Y	Y	PY	Y	Y	Y	PY	Y	Y	Y	Y
2.2 Were methods in additional to database searching used to identify relevant reports?	Y	Y	Y	Y	Y	Y	Y	PY	PY	PY	Y	Y	Y
2.3 Were the terms and structure of the search strategy likely to retrieve as many eligible studies as possible?	Y	PY	Y	Y	PY	Y	Y	PY	PY	PY	Y	Y	Y
2.4 Were restrictions based on date, publication format or language appropriate?	Y	PY	Y	PY	Y	Y	Y	Y	Y	Y	Y	Y	Y
2.5 Were efforts made to minimise error in selection of studies?	PY	Y	Y	PY	PY	Y	Y	Y	PY	PY	Y	Y	Y
Concerns regarding methods used to identify and/or select studies. Rationale for concern:	☺	☺	☺	☺	☺	☺	☺	☺	☺	☺	☺	☺	☺
**DOMAIN 3: DATA COLLECTION AND STUDY EVALUATION**													
Describe methods of data collection, what data were extracted from studies or collected through other means, how risk of bias was assessed (e.g. number of reviewers involved) and the tool used to assess risk of bias:													
3.1 Were efforts made to minimise errors in data collection?	N	Y	Y	PY	PY	Y	Y	Y	Y	Y	Y	Y	Y
3.2 Were sufficient study characteristics available for both review authors and readers to be able to interpret the results?	Y	Y	Y	PN	PY	Y	Y	Y	Y	Y	Y	Y	Y
3.3 Were all relevant study results collected for use in the synthesis?	Y	Y	Y	PN	Y	Y	Y	PY	Y	Y	Y	Y	Y
3.4 Was the risk of bias (or methodological quality) formally assessed using appropriate criteria?	NI	Y	Y	Y	NI	Y	Y	Y	PY	NI	NI	Y	Y
3.5 Were efforts made to minimise error in risk of bias assessment?	NI	Y	Y	NI	NI	Y	Y	PY	PY	PY	NI	Y	Y
Concerns regarding methods used to collect data and appraise studies. Rationale for concern.	☹	☺	☺	☹	😐	☺	☺	☺	☺	☺	☺	☺	☺

Table S6. (Continued) Assessing the quality of reviews using ROBIS tool (Tool to assess risk of bias in systematic reviews).

DOMAIN 4: SYNTHESIS AND FINDINGS	Birks, JS. 2016 [11]	Blanco-Silvente, L. et al. 2017 [18]	Bond, M. et al. 2012 [19]	Clegg, A. et al. 2002 [20]	Grimmer, T. et al. 2006 [21]	Hansen, R. A. et al. 2008 [22]	Hyde, C. et al. 2013 [23]	Kaduszkiewicz. H. et al. 2005 [24]	Kobayashi, H. et al. 2016 [25]	Livingston, G. et al. 2000 [26]	Ritchie, C. W. et al. 2004 [27]	Takeda, A. et al.2006 [28]	Trinh N. et al.2003 [29]
Describes synthesis methods:													
4.1 Did the synthesis include all studies that it should?	Y	Y	Y	PY	PY	Y	Y	Y	Y	Y	PY	Y	Y
4.2 Were all pre-defined analyses reported or departures explained?	Y	Y	Y	PN	PY	Y	Y	Y	Y	PY	Y	Y	Y
4.3 Was the synthesis appropriate given the nature and similarity in the research questions, study designs and outcomes across included studies?	Y	Y	Y	PN	PY	Y	Y	NI	Y	Y	Y	Y	Y
4.4 Was between-study variation (heterogeneity) minimal or addressed in the synthesis?	PN	Y	Y	N	PY	Y	Y	NI	Y	NI	Y	PY	NI
4.5 Were the findings robust, e.g. as demonstrated through funnel plots or sensitivity analyses?	N	Y	Y	N	PY	N	Y	NI	Y	NI	Y	PY	Y
4.6 Were biases in the primary studies minimal or addressed in the synthesis?	N	Y	Y	N	NI	N	Y	Y	Y	NI	Y	Y	Y
Concerns regarding the synthesis and findings. Reason for concern:	☹	☺	☺	☹	😐	☹	☺	😐	☺	☺	☺	☺	☺
**Phase 3. Judging risk of bias.**													
Summarize the concerns identified during the Phase 2 assessment.													
**Dominance // Concern and Ratinale for concern.**													
1. Concerns regarding specification of study eligibility criteria.	☺	☺	☺	☺	☺	☺	☺	☺	☺	☺	☺	☺	☺
2. Concerns regarding methods used to identify and/or select studies.	☺	☺	☺	☺	☺	☺	☺	☺	☺	☺	☺	☺	☺
3. Concerns regarding methods used to collect data and appraise studies	☹	☺	☺	☹	😐	☺	☺	☺	☺	☺	☺	☺	☺
4. Concerns regarding the synthesis and findings	☹	☺	☺	☹	😐	☹	☺	😐	☺	☺	☺	☺	☺

Table S6. (Continued) Assessing the quality of reviews using ROBIS tool (Tool to assess risk of bias in systematic reviews).

RISK OF BIAS IN REVIEW	Birks, JS. 2016 [11]	Blanco-Silvente, L. et al. 2017 [18]	Bond, M. et al. 2012 [19]	Clegg, A. et al. 2002 [20]	Grimmer, T. et al. 2006 [21]	Hansen, R. A. et al. 2008 [22]	Hyde, C. et al. 2013 [23]	Kaduszkiewicz. H. et al. 2005 [24]	Kobayashi, H. et al. 2016 [25]	Livingston, G. et al. 2000 [26]	Ritchie, C. W. et al. 2004 [27]	Takeda, A. et al.2006 [28]	Trinh N. et al.2003 [29]
Describe whether the conclusions were supported by evidence:													
A. Did the interpretation of the findings address all concerns identified in Domains 1 to 4?	N	Y	Y	N	PY	Y	Y	PY	Y	Y	Y	Y	Y
B. Was the relevance of identified studies to the review's research question appropiately considered?	Y	Y	Y	PY	Y	Y	Y	PY	Y	Y	Y	Y	Y
C. Did the reviewers avoid emphasizing results on the basis of their statistical significance?	N	Y	Y	PN	NI	Y	Y	Y	Y	Y	Y	Y	Y
Risk of bias in the review. Justification for risk.	☹	☺	☺	☹	😐	☺	☺	😐	☺	☺	☺	☺	☺

Y	YES
PY	PROBABLY YES
PN	PROBABLY NO
N	NO
NI	NO INFORMATION

JUSTIFICATION FOR CONCERN:		
HIGH	☹	IF ANY IS NO OR PN
LOW	☺	IF ALL ANSWERS ARE YES OR PY
UNCLEAR	😐	INSUFFICIENT DATA

SUMMARY PHASE 1:	
YES	☺
NO	☹
UNCLEAR	😐
